# A new global dataset of bioclimatic indicators

**DOI:** 10.1038/s41597-020-00726-5

**Published:** 2020-11-16

**Authors:** Sergio Noce, Luca Caporaso, Monia Santini

**Affiliations:** 1 Division on Impacts on Agriculture, Forests and Ecosystem Services (IAFES), Fondazione Centro Euro-Mediterraneo sui Cambiamenti Climatici (CMCC), Viterbo, Italy; 2 Institute of Marine Sciences (ISMAR), Centro Nazionale delle Ricerche (CNR), Rome, Italy

**Keywords:** Climate and Earth system modelling, Climate-change impacts, Environmental impact

## Abstract

This study presents a new global gridded dataset of bioclimatic indicators at 0.5° by 0.5° resolution for historical and future conditions. The dataset, called CMCC-BioClimInd, provides a set of 35 bioclimatic indices, expressed as mean values over each time interval, derived from post-processing both climate reanalysis for historical period (1960–1999) and an ensemble of 11 bias corrected CMIP5 simulations under two greenhouse gas concentration scenarios for future climate projections along two periods (2040–2079 and 2060–2099). This new dataset complements the availability of spatialized bioclimatic information, crucial aspect in many ecological and environmental wide scale applications and for several disciplines, including forestry, biodiversity conservation, plant and landscape ecology. The data of individual indicators are publicly available for download in the commonly used Network Common Data Form 4 (NetCDF4) format.

## Abstract (summary)

**Table:** 

Measurement(s)	bioclimatic indicators
Technology Type(s)	Geographic Information System • computational modeling technique
Factor Type(s)	temporal interval • geographic location
Sample Characteristic - Environment	climate system
Sample Characteristic - Location	global

Machine-accessible metadata file describing the reported data: 10.6084/m9.figshare.12927443

## Background & Summary

Climate change impacts, affecting primarily ecosystems’ functions and consequently human sectors, have become a crucial topic within the scientific community (https://www.ipcc.ch/working-group/wg2/) and most recently across the whole society and sustainable development efforts (https://sustainabledevelopment.un.org/sdg13). Average climate patterns, mainly represented by intra-annual (monthly to seasonal) temperature and precipitation cycle, directly influence the distribution, abundance and interactions of biological species, with consolidated evidence of how observed and expected variations in climate conditions can undermine the ecosystems’ ecological equilibrium^1–3^.

Under faster and faster environmental modifications over lands^4^, climate datasets and efficient processing chains applied on them allow answering many urgent questions of biogeographical sciences about climate change impacts on living organisms (e.g. animals, plants, bacteria), even through the interactions they have with the surrounding natural resources, like water and soil. For example, to reproduce and model, respectively, current and future habitats’ ranges by mean of Species Distribution Modelling (SDM), large amount of high quality and up-to-date environmental (especially climate) data is required, to be operated into further indicators proxy of typical climate settings across the domains of interest^5–9^.

During the long history of scientific research on the relationships between climate and Earth’s communities, numerous meteorological variables and/or derived indices have been formulated, calculated and applied to explain the geographic distribution of natural populations along climate gradients, characterized by intra-annual patterns of temperature and precipitation^10^. Such variables and indices are also known as bioclimatic indicators (hereafter BioClimInd). They mainly result from primary - observed or modelled - climate fields (e.g. minimum, maximum and mean temperature, precipitation amount) and contribute to delineate the bioclimatic “envelope” for species in terms of favourable environmental conditions^11,12^, also referred to as “suitability”^7,9^.

Considering the increasing interest for spatially-explicit assessments, the computation of BioClimInd for the historical period can benefit from gridded climate products building on meteorological stations’ data spatialized through either regression-based interpolation or climate reanalysis^13^. Instead, the primary data source to derive BioClimInd for future time horizons are the outputs of climate model simulations that, despite the substantial progress occurred in the last few decades, are still affected by both systematic and random errors preventing their direct use in climate impact studies without affecting their reliability^13–15^. To reduce these errors and related effects, different approaches can be used^14^: improving the physics in the models; quantifying the uncertainty by using a multi-model ensemble; or removing the model biases through climate data post-processing^16^. The first option requires an extensive investment in research efforts while the second one needs a huge computing power. Although lacking a sound physical basis, the last option, also know as “bias-correction”, has become increasingly common in the climate impact community. Thanks to its high implementation simplicity and low computational demand along with growing spatialized databases on global and regional climate observations to be used as benchmark^17^.

Under the raising availability of climate information, several global datasets of bioclimatic indicators have become popular as reference point for the scientific community. Worthy of mention are undoubtedly WorldClim^18,19^, CHELSA^20,21^, CliMond^22,23^, ecoClimate^24^, ENVIREM^25^ and MERRAclim^26^. WorldClim, the most cited dataset and recently (January 2020) updated to the 2.1 version, provides 19 BioClimInd at very high spatial resolution (up to 30 arc-seconds, ca. 0.9 km at the equator) for recent climate (1970–2000) and also for the future (2021–2040, 2041–2060, 2061–2080, 2081–2100) using 9 General Circulation and Earth System Models (GCMs and ESMs) as part of the Coupled Model Intercomparison Project Phase 6 (CMIP6). Climate time series have been downscaled, bias-corrected and covering the four Shared Socio-economic Pathways (SSPs) that drive simulations in support of the 6th Assessment Report from the Intergovernmental Panel on Climate Change (IPCC AR6) (https://pcmdi.llnl.gov/CMIP6/). CHELSA dataset (in the last version 1.2) has the same spatial resolution and indicators as WorldClim for the climatological period 1979–2013 and future periods 2041–2060 and 2061–2080 for a large set of future simulations under CMIP5 (Coupled Model Intercomparison Project Phase 5); CHELSA substantially differs from WorldClim over the mountain regions especially for rainfall-based indicators since the basic algorithm incorporates further orographic predictors^20^. Recently, still within the CHELSA project, a high resolution dataset was released with information on precipitation and temperatures from 2006 to 2100^21^. CliMond (in the latest 1.2 version), is a dataset of 40 BioClimInd for current and short to long-term future time frames from 2 GCMs with 10’ and 0.5° spatial resolution (ca. 9.2 and 55 km at the equator, respectively). While CliMond uses also radiation and pan evaporation as input variables, it is based on “old” greenhouse gas concentration scenarios (A1B and A2) supporting the IPCC 4th Assessment Report. ENVIREM provides both 16 BioClimInd at multiple spatial resolutions (up to 30 arc-seconds) from three global climate models for several periods in the past and the code to generate them for the future. EcoClimate provides data starting from Pliocene up to 2080–2100 for the same 19 BioClimInd in WorldClim but at 0.5° spatial resolution and for 9 among GCMs/ESMs from CMIP5 and PMIP3 (Paleoclimate Modelling Intercomparison Project Phase 3). MerraClim was recently added to these datasets and provides the 19 WorldClim indicators at three spatial resolutions (from 2.5 to 10 arc-minutes) for three recent decades (1980s, 1990s and 2000s) starting from hourly data of 2-meter air temperature and specific humidity (instead of precipitation). Even when the above datasets are provided interpolated to very high spatial resolution (few kilometers), the represented climate physical processes remain consistent to native map units (tens of thousands to millions of hectares), so that the datasets are more suitable to serve studies encompassing very broad domains (large regions/continents up to the entire globe) and hosting, in terms of biological organization, generalists species and ecosystems’ mosaics^27^. In case of investigations over sub-regional to local (e.g. mountain area) scale, some studies looking at species with more restricted habitats or at smaller patch ecosystems started to exploit simulations from regional climate models, like those making part of the Coordinated Regional Downscaling Experiment (CORDEX), their further downscaling and/or similar ensemble initiatives^28–31^. Moreover, aiming at CORDEX-based applications at global level would raise the issue of heterogeneity due to different combinations of global and regional models, greenhouse gas concentration scenarios and spatial resolutions (0.11°, 0.22° and/or 0.44°) across the CORDEX domains^32^. To give an example, only CORDEX domains of North America, Mediterranean and Europe are currently covered at 0.11° (ca. 12 km) resolution. Finally, CORDEX data require bias-correction as well, and only in one case (Europe domain) adjusted data have been made available to the community.

**Fig. 1:**
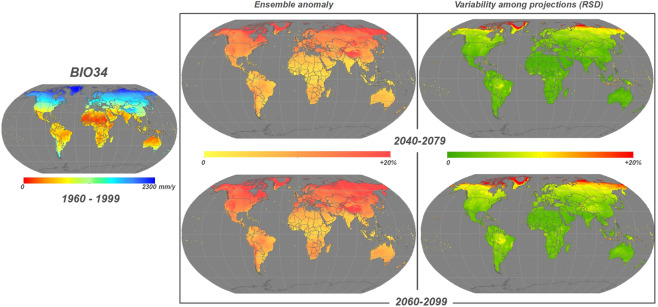
Example of BIO34 - Potential Evapotranspiration (PET, mm/y) according to Hargreaves formulation for historical time interval 1960–1999 (*left*). Ensemble anomaly of the 11 CMIP5 simulations for the future period compared to the historical one expressed in percentage (*center*) and the variation among simulation expressed in Relative Standard Deviation (RSD) (*right*) for the two time horizons 2040–2079 (*top*) and 2060–2099 (*bottom*).

**Fig. 2:**
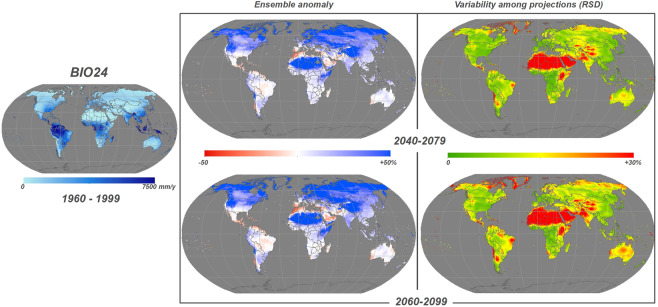
Example of BIO24 - Yearly positive precipitation (mm/y) for historical time interval 1960–1999 (*left*). Ensemble anomaly of the 11 CMIP5 simulations for the future period compared to the historical one expressed in percentage (*center*) and the variation among simulation expressed in Relative Standard Deviation (RSD) (*right*) for the two time horizons 2040–2079 (*top*) and 2060–2099 (*bottom*).

In this work, a global dataset of 35 BioClimInd is presented, named CMCC-BioClimInd, with a spatial resolution of 0.5°, both for the historical period (1960–1999) and for two future time horizons (2040–2079 and 2060–2099) from, respectively, post-processing of climate reanalysis and an ensemble of 11 CMIP5 climate simulations^33,34^. Simulations were bias-corrected benefiting of the trend-preserving approach adopted within the InterSectoral Impact-Model Intercomparison Project (ISIMIP)^35^ (see Methods). The CMCC-BioClimInd dataset contributes to widening the availability of spatial information useful to the community by (1) providing an ensemble of bioclimatic indicators for the historical and future time frames (e.g. Figs 1 and 2) (2) adopting models and/or other analysis methods for robust (i.e. taking into account uncertainty) climate change impacts’ assessments, at broad scale and in a wide range of research fields such as wildlife ecology, natural resources’ conservation and management, climate impacts’ mitigation. On the one hand, the exploitation of indicators instead of just raw climate variables enables easier inferring of relationships between the studied topic (species occurrence, resources availability etc.) and the climate regime to support decision for complex systems^36^; on the other hand, using the ensemble allows considering the variability across simulations due to the different models’ physics and the uncertain future development pathways^37^.

## Methods

### Input data

The CMCC-BioClimInd were elaborated, for the historical period, starting from the daily time series of temperature and precipitation available from the Water and Global Change (WATCH) forcing dataset (WFD) (http://www.eu-watch.org/data_availability). The WFD is a twentieth century meteorological dataset based on the European Centre for Medium-range Weather Forecasts (ECMWF) Re-Analysis (ERA-40)^38^ interpolated to a 0.5° by 0.5° grid, with successive elevation-based correction of surface meteorological variables plus monthly bias-correction from the Climatic Research Unit (CRU) gridded observational dataset^39,40^. In this dataset, the years from 1960 to 1999 were considered as reference period.

For the future time frame, daily time series of precipitation and temperature are available, thanks to the coordinated climate model simulation experiments in the frame of CMIP5^33,34^. These simulations are forced by multiple Representative Concentration Pathways (RCPs) formulated to support the IPCC AR5^41,42^. It is known, as stated above, how raw climate model simulations are however affected by biases that, to serve impact studies, need to be reduced^14^. To achieve this, the ISIMIP Fast-Track (FT) initiative (https://www.isimip.org/about/#simulation-rounds) provides bias-corrected daily time series of temperature and precipitation globally downscaled (through bilinear interpolation) at 0.5° grid resolution for five Earth System Models (ESMs) participating in CMIP5: GFDL-ESM2M, HadGEM2-ES, IPSL-CM5A-LR, MIROC-ESM-CHEM, NorESM1-M. Bias-correction is based on a trend-preserving method^35^ (https://github.com/ISIMIP/BC), developed to adjust the probability distribution over the reference period 1960–1999, while preserving the long-term trend in the data.

For the purpose of this work, this method was applied also to the ESM developed and used by the Foundation Euro-Mediterranean Center on Climate Change (CMCC-CESM^43^), and whose raw data are available on the CMIP5 data archive (https://esgf-node.llnl.gov/search/cmip5/). For the five different ESMs already bias-corrected within the ISIMIP FT initiative, the RCP4.5^44^ and the RCP8.5^45^ were selected, while projections under the RCP8.5 are available from CMCC-CESM runs.

### Bioclimatic indicators

Here, a description of BioClimInd (numbered from 1 to 35 with the prefix “Bio” and with a short name) is provided building on the definitions, in some case slightly revised, of the U.S. Geological Survey (USGS)^46^, the one in the ANUCLIM User Guide (https://fennerschool.anu.edu.au/files/anuclim61.pdf), and those in^47^ and/or^48,49^. Based on the indicator considered, source variables can be the bias-corrected mean temperature (TG), maximum temperature (TX), minimum temperature (TN) or precipitation (Pr), directly from daily series as described in the Section “Input data”. Before computing any indicator, bias-corrected daily series of maximum, minimum and mean temperature were converted from degrees Kelvin to Celsius by subtracting 273.15 and indicated as Tx, Tn and Tg, respectively, while precipitation was converted from kg/m^2^/sec to mm/day by multiplying by 86400 (seconds in a day) and the new variable is then indicated with P. The BioClimInd short name, input variables and definition sources are reported in Table 1, formulas are described in detail in Supplementary Table 1. In the extended description below, the long-term or climatological period (or simply “period”) refers to the 40-year time frames used for the analysis, i.e. 1960–1999 for the historical period (to match the reference period for the bias-correction) and 2040–2079 and 2060–2099 for the future periods, in order to enable comparison between future and historical time frames with the same length. When calendar months are mentioned, it means that a month is considered at climatological level (i.e. its 40-year average). The approach for the definition of wettest/driest/coldest/warmest months or quarters identifies these periods year by year allowing to consider inter-annual variability of intra-annual cycles, as also interestingly investigated for different “climatological” windows by^50^.

**Table 1:** Code, full names, units, main references and input variables (Daily mean *Tg*, maximum *Tx* and minimum *Tn* temperature, daily precipitation amount *P*) for the BioClimInd.

Code	Name	Unit	References	Derived from
Bio1	Annual mean temperature	°C		Tg
Bio2	Mean diurnal range	°C		Tx,Tn
Bio3	Isothermality	%		Tx,Tn
Bio4	Temperature seasonality	°C		Tg
Bio5	Max temperature of warmest month	°C		Tx
Bio6	Min temperature of coldest month	°C		Tn
Bio7	Temperature annual range	°C		Tx,Tn
Bio8	Mean temperature of wettest quarter	°C		Tg,P
Bio9	Mean temperature of driest quarter	°C		Tg,P
Bio10	Mean temperature of warmest quarter	°C		Tg
Bio11	Mean temperature of coldest quarter	°C		Tg
Bio12	Annual precipitation	mm		P
Bio13	Precipitation of wettest month	mm		P
Bio14	Precipitation of driest month	mm		P
Bio15	Precipitation seasonality	%		P
Bio16	Precipitation of wettest quarter	mm		P
Bio17	Precipitation of driest quarter	mm		P
Bio18	Precipitation of warmest quarter	mm		Tg,P
Bio19	Precipitation of coldest quarter	mm		Tg,P
Bio20	Ellenberg quotient	°C/mm	^52^	Tg,P
Bio21	Yearly positive temperature	°C	^47^	Tg
Bio22	Sum of annual temperature	°C		Tg
Bio23	Ombrotermic index	mm/°C	^47^	Tg,P
Bio24	Yearly positive precipitation	mm	^47^	Tg,P
Bio25	Modified Kira coldness index	°C	^48,49^	Tg
Bio26	Modified Kira warmth index	°C	^48,49^	Tg
Bio27	Simplified continentality index	°C	^53^	Tg
Bio28	Mean temperature of warmest month	°C		Tg
Bio29	Mean temperature of coldest month	°C		Tg
Bio30	Mean temperature of driest month	°C		Tg,P
Bio31	Mean temperature of wettest month	°C		Tg,P
Bio32	Modified Thermicity index	°C	^47^	Tg,Tx,Tn
Bio33	Ombrothermic index of summer and the previous month	mm/°C	^47^	Tg,P
Bio34	Potential Evapotranspiration Hargreaves	mm	^54^	Tg
Bio35	Potential Evapotranspiration Thornthwaite	mm	^55^	Tg

Formulas are reported in Supplementary Table 1.

- *Bio1 Annual mean temperature*

This indicator, expressed in °C, indicates the total amount of energy inputs for the ecosystems in a year. The annual average of daily mean temperature (Tg) is first computed for each year in the considered long-term period, and then it is further averaged among all years in the period.

- *Bio2 Mean diurnal range*

The daily fluctuations of temperatures have a strong influence on ecosystems^51^. This indicator is expressed in °C and is calculated by averaging, within the considered period, the daily differences between the maximum (Tx) and minimum (Tn) temperature.

- *Bio3 Isothermality*

This indicator quantifies how large the day-to-night temperatures oscillate relatively to the annual oscillations among extreme (warmest and coldest) months. Isothermality is the ratio, expressed in %, of Bio2/Bio7 (see below for Bio7).

- *Bio4 Temperature seasonality*

This indicator, expressed in °C, measures the temperature change throughout the year. Based on the formulation developed by Hijmans in WorldClim^18^, the average of daily mean temperature (Tg) is calculated for each calendar month in the selected period, and then the Standard Deviation is computed among the 12 monthly values obtained. The larger the value of the Standard Deviation, the greater the variability of temperature within the year.

- *Bio5 Maximum temperature of warmest month*

For this indicator, expressed in °C, first the monthly mean of daily maximum temperature (Tx) is calculated, then for each year in the period the warmest month, i.e. the one with the highest mean of daily Tx, is selected and the climatological mean of these values is finally computed. Note that the warmest month could be different from year to year.

- *Bio6 Minimum temperature of coldest month*

For this indicator, expressed in °C, first the monthly mean of daily minimum temperature (Tn) is calculated, then for each year in the period the coldest month, i.e. the one with the lowest mean of daily Tn, is selected and the climatological mean of these values is finally computed. Note that the coldest month could be different from year to year.

- *Bio7 Temperature annual range*

Similar to Bio2, also the temperature fluctuations between the warmest vs. coldest month within a year play a crucial role on ecosystems. This indicator measures the range of temperature between extreme (warmest and coldest) months. Practically, Bio7 is the difference between Bio5 and Bio6, expressed in °C, and is also used to calculate Bio3.

- *Bio8 Mean temperature of wettest quarter*

To calculate this indicator, expressed in °C, first the wettest quarter (i.e. that with the highest precipitation amount calculated by summing daily P) of each year in the whole period is identified (considering a quarter belonging to the year of its central month), and then the mean temperature among all wettest quarters is calculated by averaging daily Tg. Note that the wettest quarter could be different from year to year.

- *Bio9 Mean temperature of driest quarter*

To calculate this indicator, expressed in °C, first the driest quarter (i.e. that with the lowest precipitation amount calculated by summing daily P) of each year in the whole period is identified (considering a quarter belonging to the year of its central month), and then the mean temperature among all driest quarters is calculated by averaging daily Tg. Note that the driest quarter could be different from year to year.

- *Bio10 Mean temperature of warmest quarter*

To calculate this indicator, expressed in °C, first the warmest quarter (i.e. that with the highest average of daily mean temperature Tg) of each year in the whole period is identified (considering a quarter belonging to the year of its central month), and then the mean temperature among all warmest quarters is calculated by averaging daily Tg. Note that the warmest quarter could be different from year to year.

- *Bio11 Mean temperature of coldest quarter*

To calculate this indicator, expressed in °C, first the coldest quarter (i.e. that with the lowest average of daily mean temperature Tg) of each year in the whole period is identified (considering a quarter belonging to the year of its central month), and then the mean temperature among all coldest quarters is calculated by averaging daily Tg. Note that the coldest quarter could be different from year to year.

- *Bio12 Annual precipitation*

This is a widely used indicator, representing the total amount of water inputs to the ecosystems and to their water cycle. This indicator is expressed in millimeter (mm) per year and it is derived by averaging, along the whole period, the annual sum of daily precipitation amounts P.

- *Bio13 Precipitation of wettest month*

For this indicator, expressed in mm, first the monthly sum of daily precipitation P is calculated, then for each year in the period the wettest month, i.e. the one with the highest precipitation amount, is identified and then the precipitation amount among all wettest months is averaged. Note that the wettest month could be different from year to year.

- *Bio14 Precipitation of driest month*

For this indicator, expressed in mm, first the monthly sum of daily precipitation P is calculated, then for each year in the period the driest month, i.e. the one with the lowest precipitation amount, is identified and then the precipitation amount among all driest months is averaged. Note that the driest month could be different from year to year.

- *Bio15 Precipitation seasonality*

Species distribution can be heavily influenced by precipitation variability during the year^46^. The precipitation seasonality, expressed in %, is the ratio between the standard deviation and the mean of 12 values representing the monthly average precipitation as calculated at calendar month level within the considered period. To avoid division by 0, the denominator is increased by 1.

- *Bio16 Precipitation of wettest quarter*

To calculate this indicator, expressed in mm, first the wettest quarter (i.e. that with the highest precipitation amount calculated by summing daily P) of each year in the whole period is identified (considering a quarter belonging to the year of its central month) (see Bio8), and then the precipitation amount is averaged among all wettest quarters. Note that the wettest quarter could be different from year to year.

- *Bio17 Precipitation of driest quarter*

To calculate this indicator, expressed in mm, first the driest quarter (i.e. that with the lowest precipitation amount calculated by summing daily P) of each year in the whole period is identified (considering a quarter belonging to the year of its central month) (see Bio9), and then the precipitation amount is averaged among all driest quarters. Note that the driest quarter could be different from year to year.

- *Bio18 Precipitation of warmest quarter*

To calculate this indicator, expressed in mm, first the warmest quarter (i.e. that with the highest average of daily mean temperature Tg) of each year in the whole period is identified (considering a quarter belonging to the year of its central month) (see Bio10), and then the precipitation amount is averaged among all warmest quarters. Note that the warmest quarter could be different from year to year.

- *Bio19 Precipitation of coldest quarter*

To calculate this indicator, expressed in mm, first the coldest quarter (i.e. that with the lowest average of daily mean temperature Tg) of each year in the whole period is identified (considering a quarter belonging to the year of its central month) (see Bio11), and then the precipitation amount is averaged among all coldest quarters. Note that the coldest quarter could be different from year to year.

- *Bio20 Ellenberg quotient*

This indicator is firstly described by Ellenberg in 1963^52^ and it relates the temperature of the warmest month of the year to the annual precipitation. It is calculated in °C/mm as the ratio Bio28/Bio12 multiplied by 1000. This is a simple measure of humidity in respect to continentality.

- *Bio21 Yearly positive temperature*

Following the Rivas-Martinez bioclimatic approach^47^, this indicator, expressed in °C, consists in the sum of the monthly average temperature for those months whose long-term average of daily temperature Tg is higher than 0 °C.

- *Bio22 Sum of annual temperature*

This indicator is similar to Bio21 from which it differs because it is the sum of the monthly average temperature for all months expressed in °C. In large portion of the globe this data results smaller than Bio21. It is also approximately equivalent to Bio1 multiplied by 12, but has been included in this dataset for a direct comparison with Bio21, Bio25 and Bio26.

- *Bio23 Ombrothermic index*

This indicator, calculated in mm/°C, is ten times the ratio between the average precipitation amount (in mm) for months with positive average of daily Tg (Bio24) and the sum of average temperature for the same months (Bio21)^47^.

- *Bio24 Yearly positive precipitation*

Still following^47^ this indicator, expressed in mm, represents the sum of the long-term average of total precipitation (cumulated of daily P) for those months whose long-term average of daily temperature Tg is higher than 0 °C.

- *Bio25 Modified Kira coldness index*

Described by Kira^48,49^, and partially modified, this indicator describes the amount of energy inputs in the coldest portion of the year. It consists of the sum of the monthly average temperature for those months whose long-term average of daily temperature Tg is lower than 5 °C.

- *Bio26 Modified Kira warmth index*

Described by Kira^48,49^, and partially modified, this indicator describes the amount of energy inputs in the warmest portion of the year. It consists of the sum of the monthly average temperature for those months whose long-term average of daily temperature Tg is higher than 5 °C.

- *Bio27 Simplified continentality index*

Similar to Bio7, but based on Tg instead of Tx and Tn, this is an another measure of temperature extremes. It is the difference, in °C, between the average Tg of warmest (Bio28) and of the coldest (Bio29) months from the years of the considered period. Partially modified from Driscoll index^53^

- *Bio28 Mean temperature of warmest month*

For this indicator, expressed in °C, first the warmest months as in Bio5 are extracted for each year in the considered period, and then the average among daily mean temperature (Tg) is calculated among these months.

- *Bio29 Mean temperature of coldest month*

For this indicator, expressed in °C, first the coldest months as in Bio6 are extracted for each year in the considered period, and then the average among daily mean temperature (Tg) is calculated among these months.

- *Bio30 Mean temperature of driest month*

For this indicator, expressed in °C, first the driest months as in Bio14 are extracted for each year in the considered period, and then the average among daily mean temperature (Tg) is calculated among these months.

- *Bio31 Mean temperature of wettest month*

For this indicator, expressed in °C, first the wettest months as in Bio13 are extracted for each year in the considered period, and then the average among daily mean temperature (Tg) is calculated among these months.

- *Bio32 Modified Thermicity index*

Revised from what was described by Rivas-Martinez^47^, this indicator, expressed in tents of °C, is the sum of annual mean temperature (Bio1), the average of maximum temperatures of warmest month (Bio5) and the average of minimum temperatures of coldest month (Bio6).

- *Bio33 Ombrothermic index of summer and the previous month*

Modified from Rivas-Martinez^47^, this indicator, expressed in mm/°C, is suited for the Northern Hemisphere, dividing the sum of P and the average of Tg for the late spring/summer period (May, June, July and August)

- *Bio34 Potential Evapotranspiration Hargreaves*

This indicator calculates the potential evapotranspiration (PET, mm) through the formula of Hargreaves and Samani^54^, that requires as input the time series of monthly mean of Tg and of daily temperature range (this calculated from daily input of Tx and Tn). First the PET is cumulated for each year in the considered period and then averaged among years.

- *Bio35 Potential Evapotranspiration Thornthwaite*

This indicator calculates the potential evapotranspiration (PET, mm) through the formula of Thornthwaite^55^, that requires as input the time series of monthly mean of Tg. First the PET is cumulated for each year in the considered period and then averaged among years.

## Data Records

The complete CMCC-BioClimInd dataset^56^ is available through PANGAEA (10.1594/PANGAEA.904278). It consists in 805 files in NetCDF4 format with a 0.5° by 0.5° grid resolution and global coverage (except Antarctica). Files represent 35 bioclimatic indicators calculated for a 40-years historical interval (1960–1999) under climate reanalysis and for two future 40-years time intervals (2040–2079 and 2060–2099) under 6 ESMs’ projections and 2 RCPs (except RCP4.5 for CMCC-CESM) (Table 2). The size of the array for each map is 720 (longitudes) x 360 (latitudes) grid points for a total of 259200 points, whose 67415 differ from NULL as representing land areas. The individual file names follow this structure: “*BIOx institute-model rcp yyyy zz.nc*”

**Table 2:** Coverage of simulations across ESMs source of data and RCPs.

		RCP4.5		RCP8.5	
**Data Source/Period**	**1960–99**	**2040–79**	**2060–99**	**2040–79**	**2060–99**
Observations	✓				
CMCC-CESM				✓	✓
GFDL-ESM2M		✓	✓	✓	✓
HadGEM2-ES		✓	✓	✓	✓
IPSL-CM5A-LR		✓	✓	✓	✓
MIROC-ESM-CHEM		✓	✓	✓	✓
NorESM1-M		✓	✓	✓	✓

where *x* identifies the code of the bioclimatic indicator, *institute-model* (see Table 3, first column) identifies the source of data (short name for historical observation or for the producing Center and/or ESMs), *rcp* is the Representative Concentration Pathway (RCP 4.5 or RCP 8.5), *yyyy* is the starting year of time interval and *zz* the last two digits of the ending year of the time interval.

**Table 3:** Examples of NetCDF naming (fourth column) based on bioclimatic indicator code (first four of five digits, BIO1 in the example case), data source (second column as abbreviation of the first one reporting the data source name), RCP (45 for RCP 4.5 and 85 for RCP 8.5, or empty in case of the historical period under WFD data), start year of the period (four digits) and end year of the period (the last two digits of the year).

Data Source	Source short name	RCP	File naming
WFD	HIST	—	*BIO1_HIST_1960_99.nc*
CMCC-CESM	CMCC	8.5	*BIO1_CMCC_85_2060_99.nc*
GFDL-ESM2M	GFDL	8.5	*BIO1_GFDL_85_2060_99.nc*
HadGEM2-ES	HADGEM	8.5	*BIO1_HADGEM_85_2060_99.nc*
IPSL-CM5A-LR	IPSL	4.5	*BIO1_IPSL_45_2060_99.nc*
MIROC-ESM-CHEM	MIROC	4.5	*BIO1_MIROC_45_2060_99.nc*
NorESM1-M	NORESM	4.5	*BIO1_NORESM_45_2060_99.nc*

## Technical Validation

### Comparison of CMCC-BioClimInd 1.0 and WorldClim 2.0 datasets for the historical period

**Fig. 3:**
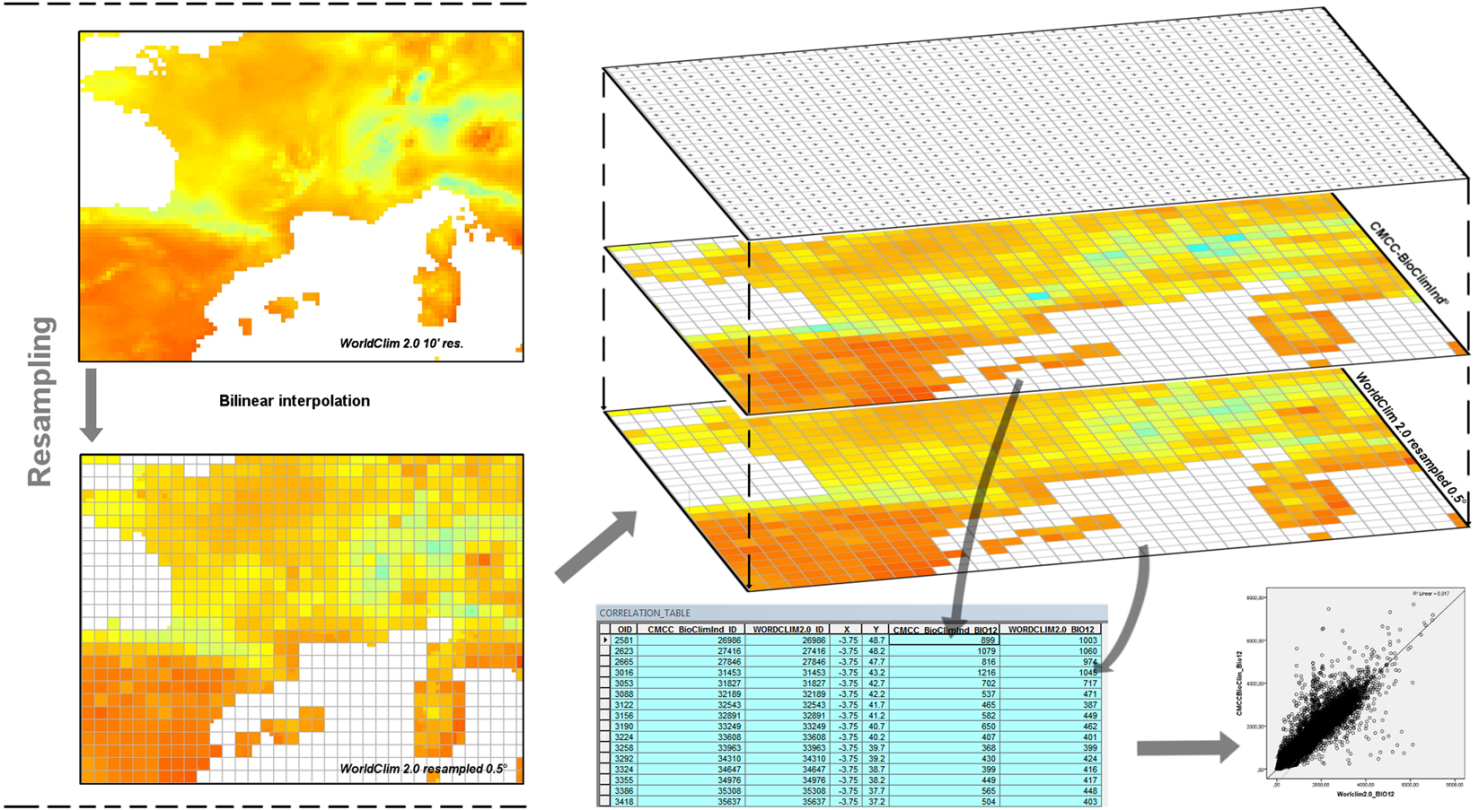
Workflow of the comparison between CMCC-BioClimInd 1.0 and WorldClim 2.0 for the historical period. The WorlClim dataset resampled to 0.5° by 0.5° grid (*left*); then overlapping of grid points from the two datasets, extraction of comparison table and creation of plots (*right*).

To check the quality of our dataset the first 19 BioClimInd were compared with the same indicators from WorldClim for the historical period, ensuring independence of datasets as relying on different climate data sources (reanalysis-based for CMCC-BioClimInd, interpolation from station data for WorldClim). The WorldClim Bioclimatic variables were downloaded from http://www.worldclim.org/ version 2 at a 10’ resolution, then resampled to a 0.5° resolution in ESRI ArcGIS Desktop 10.1 using the bilinear resampling technique (Fig. 3). Subsequently, for each indicators only the 64515 valid (different from no data) grid cells from both CMCC-BioClimInd and WorldClim datasets were considered in the comparison. It is necessary to remark that historical CMCC-BioClimInd refer to 1960–1999 period whereas WorldClim to 1970–2000.

Although the two datasets overlap in time for 30 out of 40 years (1970–1999), Fig. 4 shows a good correlation between them. For 11 BioClimInd the R^2^ is above 0.9, for 7 between 0.75 and 0.9, only the comparison of Bio19 between the two datasets has shown a lower level of correlation. In particular, for the basic indicators Annual Mean Temperature (Bio1) and Annual precipitation (Bio12) the level of correlation is excellent (i.e. R^2^ = 0.991 - 0.917).

**Fig. 4:**
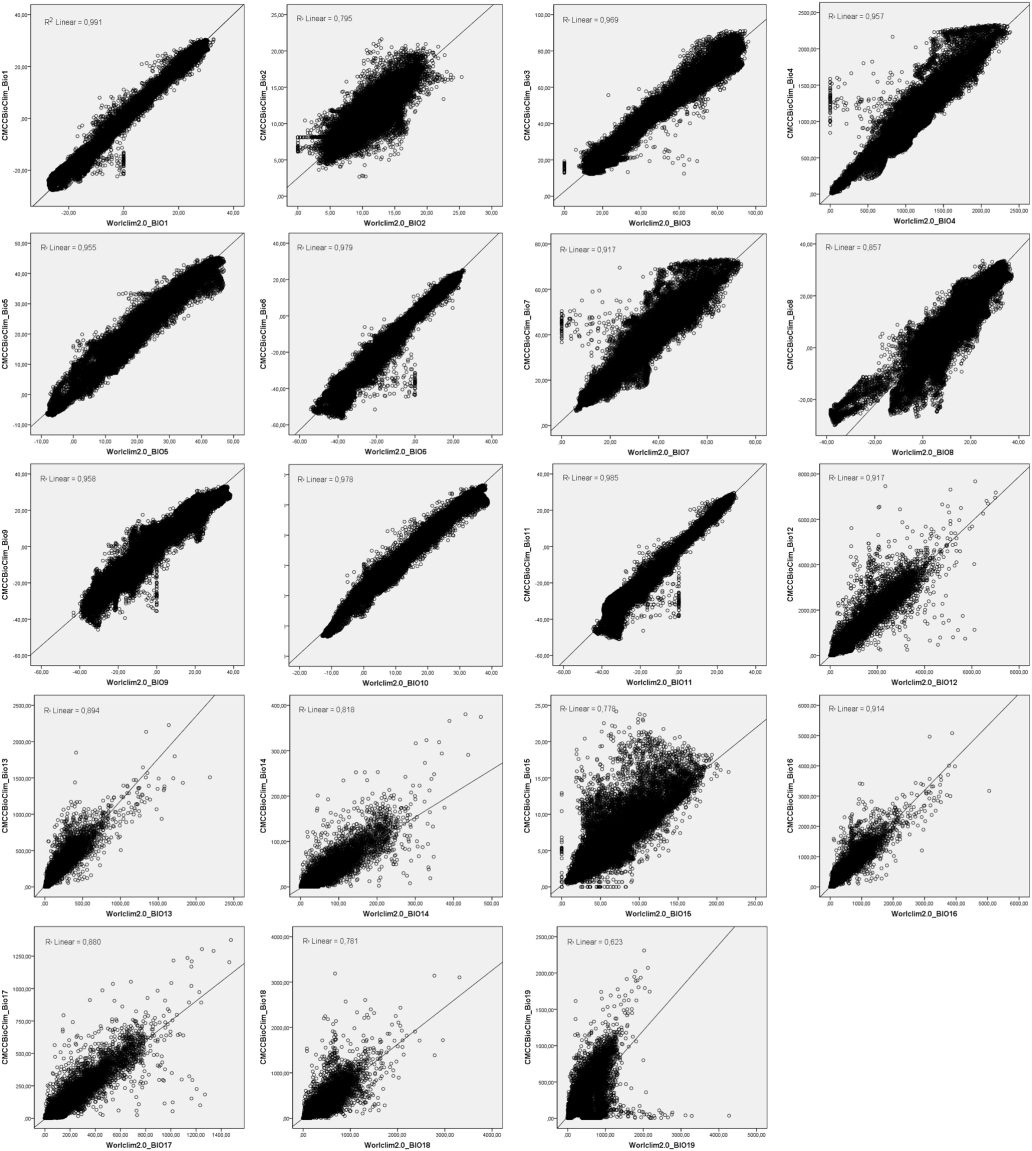
Comparison of 19 common indicators between WorldClim2.0 (x-axis) and CMCC-BioClimInd (y-axis).

**Fig. 5:**
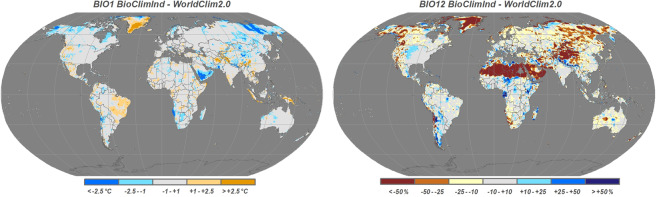
Maps of differences calculated as CMCC-BioClimInd minus WorldClim2.0. Absolute difference ( °C) for Bio1 (*left)* and percentage difference for Bio12 (*right)*.

In Fig. 5 are reported maps of spatial differences for Bio1 and Bio12; main divergences in annual temperature can be found in the North-Eastern portion of Siberia, Arabian peninsula, western Africa where BioClimInd results colder, in contrast with a large portion of Greenland and Himalayan mountains where BioClimInd results sensibly hotter. However, in most part of the globe, temperature differences are within ±2.5°C. For precipitation, the differences, in terms of lower BioCLimInd values are recognized in almost all desert areas (i.e. example Sahara, Arabian peninsula, Karakum, Gobi, Kalahari etc.) although it is important to underline that even with high percentage values the differences in absolute terms (mm) are minimal but appear high as compared to low reference values; WorlClim results sensibly drier in many areas of central Africa, Southern America and Himalayan mountains.

It is worth noticing that the above mentioned differences are mainly located in areas where the variables estimate is less accurate due to the paucity of ground observations^57^ and some artifacts may arise from the interpolation function used when creating the spatial gridded dataset. Therefore, one of the possible explanations for the differences between WorldClim and CMCC-BioClimInd, could be the different weight given to observations with respect to climate model data when creating the datasets. In Table 4 the main descriptive statistics for the 19 common indicators and the results of the Paired Samples T-Test are reported.

**Table 4:** Summary of descriptive statistics and results of Paired Samples T-Test to study the differences between CMCC-BioClimInd and WorldClim 2.0.

	Datasets descriptive statistics				Difference from WorldClim 2.0 (Paired Samples T-Test)					
	BioClimInd		WorldClim 2.0		Mean	St.Dev	99% Confidence Interval of the Difference		t(df = 64514)	p-value
	*Mean*	*St.Dev*	*Mean*	*St.Dev*			*Lower*	*Upper*		
Bio1	8.126	15.097	8.350	14.839	−0.223	1.463	−0.238	−0.209	−38.782	<0.0001
Bio2	11.077	2.862	10.855	3.234	0.222	1.466	0.006	0.207	38.492	<0.0001
Bio3	37.675	19.560	38.995	21.426	−1.312	4.062	−1.361	−1.279	−82.531	<0.0001
Bio4	835,284	534.812	848.365	528.491	−13.080	110.913	−14.205	−11.955	−29.955	<0.0001
Bio5	26.222	10.350	24.987	10.493	1.235	2.223	1.212	1.257	141.086	<0.0001
Bio6	−9.506	21.000	−8.984	19.869	−0.522	3.167	−0.554	−0.491	−41.878	<0.0001
Bio7	35.728	14.274	33.971	13.830	1.757	4.115	1.715	1.799	108.452	<0.0001
Bio8	13.074	12.106	15.189	11.266	−2.115	4.571	−2.161	−2.068	−117.507	<0.0001
Bio9	3.937	19.139	2.933	20.174	1.003	4.200	0.961	1.046	60.698	<0.0001
Bio10	18.627	9.784	18.707	9.899	−0.801	1.454	−0.095	−0.653	−13.987	<0.0001
Bio11	−2.832	20.955	−1.944	20.006	−0.886	2.653	−0.914	−0.860	−84.965	<0.0001
Bio12	682.890	709.593	711.460	687.841	−28.560	204.944	−30.639	−26.482	−35.396	<0.0001
Bio13	153.302	138.876	117.571	112.562	35.731	48.866	35.235	36.226	185.722	<0.0001
Bio14	8.139	18.342	20.837	31.739	−12.697	17.058	−12.870	−12.524	−189.066	<0.0001
Bio15*	55.381	32.141	61.408	32.726	−55.870	29.929	−6.027	15.748	−97.204	<0.0001
Bio16	338.706	332.544	311.413	299.049	27.293	99.232	26.287	28.299	69.860	<0.0001
Bio17	49.865	85.097	73.531	105.357	−23.665	38.987	−24.061	−23.270	−154.179	<0.0001
Bio18	175.365	176.338	212.796	191.427	−37.432	89.900	−38.342	−36.521	−105.875	<0.0001
Bio19	191.427	158.270	132.108	211.287	−28.617	130.071	−29.936	−27.298	−55.883	<0.0001

*BioClimInd Bio15 * *10*.

### Further remarks

After the above necessary comparison, the authors want to emphasize that the entire dataset presented in this work is based on elaborations of climate reanalysis for the historical period, whose validation effort are available in literature^40,58,59^. Hence, it can be assumed that outputs are at least as robust as the datasets from which they originate and therefore do not need further validation. For future projections, the dataset uses bias-corrected outputs of climate models and it is recommended to exploit this dataset under an ensemble perspective^37^. In conclusion it should be emphasized that combining several parameters for the creation of indices (see Table 1) inevitably increases the level of uncertainty^16^ due to error propagation.

## Usage Notes

When using CMCC-BioclimInd dataset or part of it, please cite this manuscript. Besides data sources for the dataset presented in this manuscript, data from further climate simulations can be integrated, e.g. considering also RCPs 2.6 and 6.0 and/or additional models as available from other ISIMIP rounds. Data are in NetCDF4 format, but other common interoperable formats (i.e. ESRI grid, GEOTIFF) can be provided, by contacting the authors, for a sub-selection of the dataset, whose size will be defined based on the specific requests from interested users and on the processing time needed. For any questions, suggestions or request of collaboration regarding CMCC-BioClimInd please contact the corresponding author. Data are freely available under the Creative Commons License: CC BY

## Supplementary information

Supplementary Table 1

## Data Availability

All the BioClimInd calculations were conducted exploiting NetCDF data manipulation and analysis tools, namely CDO (Climate Data Operators) and NCO (NetCDF Operators) combined through DOS Batch (.bat) commands and scripts available through Github at https://github.com/CMCC-Foundation/BioClimInd.
