# Supplementary material for: A new global dataset of bioclimatic indicators
Source: Sci Data. 2020 Nov 16;7:398. doi: 10.1038/s41597-020-00726-5 (PMC7670417; doi:10.1038/s41597-020-00726-5)
Supplement: Supplementary file 1 — Supplementary Table 1 [file 41597_2020_726_MOESM1_ESM.docx]

**Supplementary Table 1 : Formulation of BioClimInd**

**Symbols**

$\forall$ for any

$\in$ belonging to

Tg=daily mean temperature in °C

Tx=daily maximum temperature in °C

Tn=daily minimum temperature in °C

P=daily precipitation amount in mm

d=day index (1 to D_m_ for months, 1 to D_y_ for years, 1 to D_q_ for quarters)

m=month index (1 to 12)

q=quarter index (1 to 12 as running quarters are considered, for each year those with a least two out of three months belonging to the same year; i.e. December, January, February; January, February, March; etc.)

y=year index (1 to 40 in the present case)

Y=total number of years in a period (40 in the present case)

M=total number of months in a year (12)

D_y_=total number of days in the year y (365 or 366 for leap years)

D_m_=total number of days in the month m

D_m,y_=total number of days in the specific month m of year y

D_q_=total number of days in the quarter q

Weq=wettest quarter

Drq=driest quarter

Waq=warmest quarter

Coq=coldest quarter

D_Weq_=total number of days in the wettest quarter

D_Drq_=total number of days in the driest quarter

D_Waq_=total number of days in the warmest quarter

D_Coq_=total number of days in the coldest quarter

Wem=wettest month

Drm=driest month

Wam=warmest month

Com=coldest month

D_Wem_=total number of days in the wettest month

D_Drm_=total number of days in the driest month

D_Wam_=total number of days in the warmest month

D_Com_=total number of days in the coldest month

Gt0=greater than 0°C

Gt5=greater than 5°C

Lt5=lesser than 5°C

$\bar{Tg}$=average temperature among all calendar months (i.e. all months of all years)

$\bar{P}$=average precipitation among all calendar months (i.e. all months of all years)

| **Bio** | **Name (units) [reference]** | **Formulation** |
| --- | --- | --- |
| Bio1 | Annual mean temperature (°C) | $\frac{1}{Y}\sum_{y=1}^{Y} \left( \frac{1}{D_{y}}\sum_{d=1}^{D_{y}} Tg \right)$ |
| Bio2 | Mean diurnal range (°C) | $\frac{1}{Y}\sum_{y=1}^{Y} \left( \frac{1}{D_{y}}\sum_{d=1}^{D_{y}} \left( Tx-Tn \right) \right)$ |
| Bio3 | Isothermality (%) | $100*\frac{Bio2}{Bio7}$ |
| Bio4 | Temperature seasonality (°C) | $\sqrt{\frac{\sum_{m=1}^{M} \left[ \left( \frac{1}{Y}\sum_{y=1}^{Y} \left( \frac{1}{D_{m,y}}\sum_{d=1}^{D_{m,y}} Tg \right) \right)-\bar{Tg} \right]^{2}}{M}}$  *where*  $\bar{Tg}$=$\frac{1}{M}\sum_{m=1}^{M} \left( \frac{1}{Y}\sum_{y=1}^{Y} \left( \frac{1}{D_{m,y}}\sum_{d=1}^{D_{m,y}} Tg \right) \right)$ |
| Bio5 | Maximum temperature of warmest month (°C) | $\frac{1}{Y}\sum_{y=1}^{Y} \left[ \max_{1\leq m\leq12}\left( \frac{1}{D_{m,y}}\sum_{d=1}^{D_{m,y}} Tx \right) \right]$ |
| Bio6 | Minimum temperature of coldest month (°C) | $\frac{1}{Y}\sum_{y=1}^{Y} \left[ \min_{1\leq m\leq12}\left( \frac{1}{D_{m,y}}\sum_{d=1}^{D_{m,y}} Tn \right) \right]$ |
| Bio7 | Temperature annual range (°C) | $Bio5-Bio6$ |
| Bio8 | Mean Temperature of the wettest quarter (°C) | $\frac{1}{Y}\sum_{y=1}^{Y} {Tg}_{Weq\left( y \right)} where \forall y\in Y$  $Weq\left( y \right) is that corresponding to\max_{1\leq q\leq12}\left( \sum_{d=1}^{D_{q}} P \right)$  ${Tg}_{Weq(y)}=\frac{1}{D_{Weq(y)}}\sum_{d=1}^{D_{Weq(y)}} Tg$ |
| Bio9 | Mean Temperature of the driest quarter (°C) | $\frac{1}{Y}\sum_{y=1}^{Y} {Tg}_{Drq\left( y \right)} where \forall y\in Y$  $Drq\left( y \right) is that corresponding to\min_{1\leq q\leq12}\left( \sum_{d=1}^{D_{q}} P \right)$  ${Tg}_{Drq(y)}=\frac{1}{D_{Drq(y)}}\sum_{d=1}^{D_{Drq(y)}} Tg$ |
| Bio10 | Mean Temperature of the warmest quarter (°C) | $\frac{1}{Y}\sum_{y=1}^{Y} {Tg}_{Waq\left( y \right)} where \forall y\in Y$  $Waq\left( y \right) is that corresponding to\max_{1\leq q\leq12}\left( \frac{1}{D_{q}}\sum_{d=1}^{D_{q}} Tg \right)$  ${Tg}_{Waq(y)}=\frac{1}{D_{waq(y)}}\sum_{d=1}^{D_{Waq(y)}} Tg$ |
| Bio11 | Mean Temperature of the coldest quarter (°C) | $\frac{1}{Y}\sum_{y=1}^{Y} {Tg}_{Coq\left( y \right)} where \forall y\in Y$  $Coq\left( y \right) is that corresponding to\min_{1\leq q\leq12}\left( \frac{1}{D_{q}}\sum_{d=1}^{D_{q}} Tg \right)$  ${Tg}_{Coq(y)}=\frac{1}{D_{Coq(y)}}\sum_{d=1}^{D_{Coq(y)}} Tg$ |
| Bio12 | Annual precipitation (mm) | $\frac{1}{Y}\sum_{y=1}^{Y} \left( \sum_{d=1}^{D_{y}} P \right)$ |
| Bio13 | Precipitation of wettest month (mm) | $\frac{1}{Y}\sum_{y=1}^{Y} P_{Wem\left( y \right)} where \forall y\in Y$  $Wem\left( y \right) is that corresponding to\max_{1\leq m\leq12}\left( \sum_{d=1}^{D_{m}} P \right)$  $P_{Wem(y)}=\sum_{d=1}^{D_{Wem(y)}} P$ |
| Bio14 | Precipitation of driest month (mm) | $\frac{1}{Y}\sum_{y=1}^{Y} P_{Drm\left( y \right)} where \forall y\in Y$  $Drm\left( y \right) is that corresponding to\min_{1\leq m\leq12}\left( \sum_{d=1}^{D_{m}} P \right)$  $P_{Drm(y)}=\sum_{d=1}^{D_{Drm(y)}} P$ |
| Bio15 | Precipitation seasonality (%) | $100 x \frac{\sqrt{\frac{\sum_{m=1}^{M} \left[ \left( \frac{1}{Y}\sum_{y=1}^{Y} \left( \sum_{d=1}^{D_{m,y}} P \right) \right)-\bar{P} \right]^{2}}{M}}}{\bar{P}+1}$  *where*  $\bar{P}$=$\frac{1}{M}\sum_{m=1}^{M} \left( \frac{1}{Y}\sum_{y=1}^{Y} \left( \sum_{d=1}^{D_{m,y}} P \right) \right)$ |
| Bio16 | Precipitation of wettest quarter (mm) | $\frac{1}{Y}\sum_{y=1}^{Y} P_{Weq\left( y \right)} where \forall y\in Y$  $Weq\left( y \right) is that corresponding to\max_{1\leq q\leq12}\left( \sum_{d=1}^{D_{q}} P \right)$  $P_{Weq(y)}=\sum_{d=1}^{D_{Weq(y)}} P$ |
| Bio17 | Precipitation of driest quarter (mm) | $\frac{1}{Y}\sum_{y=1}^{Y} P_{Drq\left( y \right)} where \forall y\in Y$  $Drq\left( y \right) is that corresponding to\min_{1\leq q\leq12}\left( \sum_{d=1}^{D_{q}} P \right)$  $P_{Drq(y)}=\sum_{d=1}^{D_{Drq(y)}} P$ |
| Bio18 | Precipitation of warmest quarter (mm) | $\frac{1}{Y}\sum_{y=1}^{Y} P_{Waq\left( y \right)} where \forall y\in Y$  $Waq\left( y \right) is that corresponding to\max_{1\leq q\leq12}\left( \frac{1}{D_{q}}\sum_{d=1}^{D_{q}} Tg \right)$  $P_{Waq(y)}=\sum_{d=1}^{D_{Waq(y)}} P$ |
| Bio19 | Precipitation of coldest quarter (mm) | $\frac{1}{Y}\sum_{y=1}^{Y} P_{Coq\left( y \right)} where \forall y\in Y$  $Coq\left( y \right) is that corresponding to\min_{1\leq q\leq12}\left( \frac{1}{D_{q}}\sum_{d=1}^{D_{q}} Tg \right)$  $P_{Coq(y)}=\sum_{d=1}^{D_{Coq(y)}} P$ |
| Bio20 | Ellenberg quotient (°C/mm) *[43]* | $1000*\frac{Bio28}{Bio12}$ |
| Bio21 | Yearly positive temperature (°C) *[40]* | $\sum_{m=1}^{M} T_{gt0\left( m \right)} where \forall m\in M$  $T_{gt0\left( m \right)}=\frac{1}{Y}\sum_{y=1}^{Y} \left( \frac{1}{D_{m,y}}\sum_{d=1}^{D_{m,y}} Tg \right) if \frac{1}{Y}\sum_{y=1}^{Y} \left( \frac{1}{D_{m,y}}\sum_{d=1}^{D_{m,y}} Tg \right)>0^{\circ}C$  $else$  $T_{gt0\left( m \right)}=0$ |
| Bio22 | Sum of annual temperature (°C) | $\frac{1}{Y}\sum_{y=1}^{Y} \left( \sum_{m=1}^{M} \left( \frac{1}{D_{m}}\sum_{d=1}^{D_{m}} Tg \right) \right)$ |
| Bio23 | Ombrothermic index (mm/°C) *[40]* | $10*\frac{Bio24}{Bio21}$ |
| Bio24 | Yearly positive precipitation (mm) *[40]* | $\sum_{m=1}^{M} P_{gt0\left( m \right)} where \forall m\in M$  $P_{gt0\left( m \right)}=\frac{1}{Y}\sum_{y=1}^{Y} \left( \sum_{d=1}^{D_{m,y}} P \right) if \frac{1}{Y}\sum_{y=1}^{Y} \left( \frac{1}{D_{m,y}}\sum_{d=1}^{D_{m,y}} Tg \right)>0^{\circ}C$  $else$  $P_{gt0\left( m \right)}=0$ |
| Bio25 | Modified Kira coldness index (°C) *[41,42]* | $\sum_{m=1}^{M} T_{lt5\left( m \right)} where \forall m\in M$  $T_{lt5\left( m \right)}=\frac{1}{Y}\sum_{y=1}^{Y} \left( \frac{1}{D_{m,y}}\sum_{d=1}^{D_{m,y}} Tg \right) if \frac{1}{Y}\sum_{y=1}^{Y} \left( \frac{1}{D_{m,y}}\sum_{d=1}^{D_{m,y}} Tg \right)<5^{\circ}C$  $else$  $T_{lt5\left( m \right)}=0$ |
| Bio26 | Modified Kira warmth index (°C) *[41,42]* | $\sum_{m=1}^{M} T_{gt5\left( m \right)} where \forall m\in M$  $T_{gt5\left( m \right)}=\frac{1}{Y}\sum_{y=1}^{Y} \left( \frac{1}{D_{m,y}}\sum_{d=1}^{D_{m,y}} Tg \right) if \frac{1}{Y}\sum_{y=1}^{Y} \left( \frac{1}{D_{m,y}}\sum_{d=1}^{D_{m,y}} Tg \right)>5^{\circ}C$  $else$  $T_{gt5\left( m \right)}=0$ |
| Bio27 | Simplified continentality index (°C) *[45,46]* | $Bio28-Bio29$ |
| Bio28 | Mean temperature of warmest month (°C) | $\frac{1}{Y}\sum_{y=1}^{Y} {Tg}_{Wam\left( y \right)} where \forall y\in Y$  $Wam\left( y \right) is that corresponding to\max_{1\leq m\leq12}\left( \frac{1}{D_{m}}\sum_{d=1}^{D_{m}} Tg \right)$  ${Tg}_{Wam(y)}=\frac{1}{D_{Wem(y)}}\sum_{d=1}^{D_{Wam(y)}} Tg$ |
| Bio29 | Mean temperature of coldest month (°C) | $\frac{1}{Y}\sum_{y=1}^{Y} {Tg}_{Wam\left( y \right)} where \forall y\in Y$  $Com\left( y \right) is that corresponding to\min_{1\leq m\leq12}\left( \frac{1}{D_{m}}\sum_{d=1}^{D_{m}} Tg \right)$  ${Tg}_{Com(y)}=\frac{1}{D_{Com(y)}}\sum_{d=1}^{D_{Com(y)}} Tg$ |
| Bio30 | Mean temperature of driest month (°C) | $\frac{1}{Y}\sum_{y=1}^{Y} {Tg}_{Drm\left( y \right)} where \forall y\in Y$  $Drm\left( y \right) is that corresponding to\min_{1\leq m\leq12}\left( \sum_{d=1}^{D_{m}} P \right)$  ${Tg}_{Drm(y)}=\frac{1}{D_{Drm(y)}}\sum_{d=1}^{D_{Drm(y)}} Tg$ |
| Bio31 | Mean temperature of wettest month (°C) | $\frac{1}{Y}\sum_{y=1}^{Y} {Tg}_{Wem\left( y \right)} where \forall y\in Y$  $Wem\left( y \right) is that corresponding to\max_{1\leq m\leq12}\left( \sum_{d=1}^{D_{m}} P \right)$  ${Tg}_{Wem(y)}=\frac{1}{D_{Wem(y)}}\sum_{d=1}^{D_{Wem(y)}} Tg$ |
| Bio32 | Modified Thermicity index (°C x 10) *[40]* | $10* \left( Bio1+Bio5+Bio6 \right)$ |
| Bio33 | Ombrothermic index of summer and the previous month (mm/°C) *[40]* | $\frac{\frac{1}{Y}\sum_{y=1}^{Y} \left( \sum_{m=5}^{8} \left( \sum_{d=1}^{D_{m,y}} P \right) \right)}{\frac{1}{Y}\sum_{y=1}^{Y} \left( \frac{1}{4}\sum_{m=5}^{8} \left( \sum_{d=1}^{D_{m,y}} Tg \right) \right)}$ |
| Bio34 | Potential Evapotranspiration Hargreaves (mm) | See *[47]* |
| Bio35 | Potential Evapotranspiration Thornthwaite (mm) | See *[48]* |
